# Evolutionary, Neural, or LLM-Driven Heuristic Generation? A Unified Ant Colony Optimization Benchmark for Nature-Inspired Routing Heuristics on the TSP and CVRP

**DOI:** 10.3390/biomimetics11070516

**Published:** 2026-07-22

**Authors:** Haoyuan Wu, You Wu

**Affiliations:** 1 School of Finance, Jiangxi University of Finance and Economics, Nanchang 330013, China; 2202301673@stu.jxufe.edu.cn; 2 Software College, Northeastern University, Shenyang 110169, China

**Keywords:** biomimetics, stigmergy, evolutionary computation, genetic programming hyper-heuristics, ant colony optimization, swarm intelligence, large language models

## Abstract

Biomimetic optimization transfers biological information-processing mechanisms into computational systems. Ant colony optimization (ACO) is a canonical example: artificial ants functionally abstract pheromone-mediated stigmergy, decentralized exploration, trail decay through algorithmic evaporation, and adaptive path reinforcement. Building on this functional biological analogue, we present a controlled cross-paradigm evaluation of routing-heuristic generation. A standardized interface embeds human-designed rules, the genetic programming hyper-heuristic GHPP, a resource-constrained DeepACO-MLP proxy, and an offline ReEvo-style proxy into the same ACO solver. The methods are evaluated on held-out TSP and CVRP instances in terms of solution quality, reported generation or training cost, interpretability, and cross-scale behavior under a matched distribution. GHPP yields the shortest routes at all tested scales; the ReEvo-offline proxy and strong human-designed rules generally form a second tier, whereas the resource-constrained neural proxy degrades markedly as problem size increases. These results do not establish an intrinsic ranking of full-capability paradigms. Instead, they show that method selection depends on the operating constraint and on evidence provenance: longer locally measured offline search favors GHPP, while auditable explicit rules characterize the human and ReEvo-offline proxies. By holding the ant-inspired execution mechanism fixed and varying the source of heuristic information, the benchmark clarifies how evolutionary, neural, and LLM-style design strategies interact with a common biomimetic substrate.

## 1. Introduction

Combinatorial optimization lies at the intersection of operations research and artificial intelligence, and nature-inspired methods have long been one of its most productive sources of practical algorithms. The traveling salesman problem (TSP) and the capacitated vehicle routing problem (CVRP) arise widely in logistics, route planning, production scheduling, and communication-network resource allocation. Exact solvers such as Concorde [1] and strong heuristics such as HGS-CVRP [2] perform well on small and medium instances, but as instance size and operational constraints grow, exact solving and manual tuning struggle to satisfy solution quality, computational time, and interpretability at once. This motivates methods that generate heuristics automatically.

Biomimetics transfers functions, mechanisms, and organizational principles from biological systems to technological design [3]. In computational optimization, this transfer concerns information-processing strategies as well as physical morphology. Social ants discover and reinforce efficient foraging paths without centralized control: individuals deposit and follow pheromone trails, and colony-level route selection emerges through repeated local positive feedback [4]. In ACO, probabilistic route construction, designed pheromone evaporation, and repeated reinforcement abstract this stigmergic principle into a practical engineering search mechanism [5].

In the present benchmark, biomimetics is therefore not merely a descriptive label but the common execution layer. Every compared method supplies local desirability information ηij to the same implemented ACO route-construction and pheromone-update interface. The paradigms differ in how they generate ηij—human knowledge, genetic evolution, neural learning, or LLM-style reflective evolution—but their decisions are expressed through a fixed, ant-inspired feedback loop. This design isolates how alternative artificial adaptation mechanisms interact with a shared biological analogue and makes their engineering trade-offs directly inspectable.

Neural combinatorial optimization (NCO) replaces part of the manual heuristic design with learning. Reinforcement learning [6], pointer networks [7], attention-based models [8], and POMO [9] advanced routing-problem solving. The original DeepACO [10] uses graph neural networks within ACO to learn heuristic information; the present study evaluates only a resource-constrained MLP proxy of that interface. LEHD [11] targets cross-scale generalization. As the survey by Wu et al. [12] notes, NCO still faces limitations in large-scale generalization, data efficiency, and fair comparison with traditional methods.

A complementary line, evolutionary hyper-heuristics, searches the space of heuristic rules rather than solving individual instances [13,14]. GHPP [15] shows that genetic programming, a core evolutionary computation technique, can generate competitive rules without deep-model training. Most recently, large language models (LLMs) have opened a new frontier of evolutionary computation: AEL [16], FunSearch [17], EoH [18], ReEvo-offline proxy [19], and MEoH [20] use LLMs as learned variation operators that generate heuristics through code generation, reflective feedback, and evolutionary search.

These paradigms, however, are still evaluated within their own conventions, under different instance distributions, solver parameters, and metrics. A central question therefore remains open: under controlled variables, which heuristic-generation paradigm best fits the quality, cost, and interpretability trade-off in practice? Three barriers have kept it unanswered. First, output heterogeneity: expression trees (GHPP), network weights (DeepACO-MLP proxy), code functions (ReEvo-offline proxy), and fixed formulas (human rules) cannot be compared directly. Second, fairness pitfalls: each original study uses different instance scales, training-data sizes, and ACO hyperparameters, so reproducing the original settings is not a fair comparison. Third, missing dimensions: most studies report only average tour length, leaving search cost, interpretability, and cross-scale stability unquantified. To address these issues, we design a standardized heuristic adapter that maps heterogeneous outputs onto one ACO solver, and we compare human-designed heuristics, GHPP, DeepACO-MLP proxy, and ReEvo-offline proxy on the same instances, the same solver, and a four-dimensional metric system.

Table 1 summarizes the resulting research gaps, the corresponding research questions, and how this paper addresses each.

Our contributions are threefold. (1) Methodologically, we provide a common ACO execution interface for heterogeneous heuristic representations and document the adapter, feasibility masks, pheromone updates, and random-seed protocol. (2) Empirically, we report a corrected conditional comparison: GHPP has the best route quality in the archived runs; strong human rules and the ReEvo-offline proxy form a second tier; and the resource-constrained DeepACO-MLP proxy should not be generalized to the original GNN. (3) Reproducibly, we separate generation/training from solver-evaluation time, distinguish local measurements from source-study estimates, correct the four-scale mean gaps, and publish the code/configuration commit used for the audit.

## 2. Related Work and SOTA Positioning

### 2.1. Traditional Heuristics and Exact Solving Methods

Traditional combinatorial optimization methods can be divided into exact solving, human-designed heuristics, and metaheuristics. Exact solving is represented by branch-and-bound, cutting planes, and branch-and-cut, and can provide proofs of optimality, but the computational burden increases rapidly as problem size grows. Human-designed heuristics construct feasible solutions through domain rules. For example, the savings algorithm proposed by Clarke and Wright [21] and the local-search strategy proposed by Lin and Kernighan [22] have long retained practical value in vehicle routing and TSP. The ant colony system proposed by Dorigo and Gambardella [5] further realizes population-based search through pheromone mechanisms, providing the methodological basis for the use of a unified ACO solver in this paper.

### 2.2. Neural Combinatorial Optimization Methods

NCO methods emphasize learning construction or improvement strategies from data. Bello et al. [6], Vinyals et al. [7], Kool et al. [8], and Kwon et al. [9] advanced routing-problem solving from the perspectives of reinforcement learning, sequence modeling, self-attention, and multi-start policy optimization, respectively. DeepACO-MLP proxy retains the ACO framework while using neural networks to learn heuristic information, thereby reducing the burden of hand-crafted design [10]. LEHD targets cross-scale generalization through a light-encoder and heavy-decoder architecture [11]. These methods represent mainstream progress in learning-based solvers, but their performance is usually affected by the training distribution, data scale, and GPU training cost.

### 2.3. Evolutionary Hyper-Heuristics and LLM-Driven Automated Algorithm Design

Evolutionary hyper-heuristics do not directly optimize a single instance; instead, they search the heuristic space for transferable rules. GHPP uses genetic programming to evolve rule expressions and obtains interpretable heuristic structures at relatively low computational cost [15]. The recent frontier extends this evolutionary perspective by combining LLMs with evolutionary computation: AEL, EoH, and MEoH treat the LLM as an intelligent variation operator, extending the boundary of automated algorithm design from the perspectives of algorithm-level evolution, idea–code co-evolution, and multi-objective heuristic search [16,18,20]. ReEvo-offline proxy combines LLM-generated code with evolutionary feedback through reflective evolution and can generate readable heuristic rules with limited human intervention [19]. Conceptually, GHPP and ReEvo-offline proxy therefore represent two points on a continuum of evolutionary heuristic generation: symbolic genetic programming and LLM-driven reflective evolution.

More recent LLM-based automatic heuristic design has expanded beyond code-first reflective evolution. Knowledge-first or top-down AHD treats reusable design principles as primary search objects and uses executable code to instantiate and test them [23]. PathWise formulates heuristic evolution as state-aware multi-agent planning through policy, world-model, and critic agents [24]. MoH uses meta-optimization to construct downstream heuristic optimizers across tasks [25], while Clade-AHD replaces node-level MCTS estimates with clade-level Bayesian beliefs and Thompson sampling under limited evaluation budgets [26]. These developments primarily alter the search controller, memory, or optimizer-generation process. Accordingly, the present ReEvo-offline proxy is treated as one reflective-evolution-style comparator, not as an exhaustive representation of current LLM-AHD systems.

### 2.4. Comparative Positioning Against Recent SOTA Methods

To clarify the relationship with recent SOTA methods, this paper divides related methods into two categories. The first category consists of heuristic-generation methods directly included in the unified experimental framework, such as DeepACO-MLP proxy, GHPP, and ReEvo-offline proxy. The second category consists of high-performance solvers used as external benchmarks or for methodological positioning, such as Concorde, HGS-CVRP, and LEHD. The core objective of this paper is not simply to pursue the shortest possible path, but to compare the relative trade-offs among different heuristic-generation paradigms in terms of solution quality, computational cost, interpretability, and small-sample conditions. Table 2 presents the SOTA positioning adopted in this paper.

Nature-inspired and hybrid metaheuristics are also applied beyond routing. An unpublished M.S. thesis reports a hybrid heuristic–metaheuristic framework for operating-room scheduling in veterinary medicine [27]; artificial bee colony optimization with nonlinear pushover analysis has been used for reinforced-concrete wall-frame design [28]; and ACO has similarly been coupled with nonlinear pushover analysis for steel chevron-braced frames [29]. These studies illustrate the cross-domain reach of bio-inspired metaheuristics and motivate evaluation frameworks that account for application structure and evaluation cost. They motivate future extensions of the present interface, but they are not treated as empirical evidence from the current TSP/CVRP experiments.

## 3. Materials and Methods

### 3.1. Problem Definition

#### 3.1.1. Traveling Salesman Problem

Given a set of city coordinates C = {c_1_, c_2_, …, c_n_} with c_i_ = (x_i_, y_i_) ∈ [0,1]^2^, the TSP aims to find the shortest closed tour that visits each city exactly once and returns to the starting city. The objective function is formulated as follows:

(1) π* = arg min(π ∈ S_n_) L(π),     L(π) = ∑_i=1_^n^ d(c(π_i_), c(π_i+1_)),    π_n+1_ = π_1_

where π denotes the visiting order, d(·,·) denotes the Euclidean distance, and S_n_ denotes the feasible solution space consisting of all city permutations.

#### 3.1.2. Capacitated Vehicle Routing Problem

CVRP adds vehicle-capacity constraints to the TSP. Given a depot, a customer set C, customer demands q_i_, a vehicle set K, and vehicle capacity Q, the objective is to minimize the total route length while satisfying all customer demands and ensuring that the load of each vehicle does not exceed its capacity.

(2) $$\min\sum_{k\in K}\sum_{i\in V}\sum_{j\in V}d_{ij}\;x_{ijk},\;s.t.\;\sum_{i\in C}q_{i}\;y_{ik}\leq Q,\;\forall k\in K$$

where x_ijk_ indicates whether vehicle k travels from node i to node j and y_ik_ indicates whether customer i is served by vehicle k.

### 3.2. Unified ACO Evaluation Framework

To avoid bias caused by different solver implementations, all heuristic-generation methods in this paper are connected to the same ACO solver. The state-transition probability adopts a weighted product of pheromone information and heuristic information:

(3) $$p_{ij}^{k}=\frac{{\left[\tau_{ij}\right]}^{\alpha }\cdot {\left[\eta_{ij}\right]}^{\beta }}{\sum_{l\in N\left(i,k\right)}{\left[\tau_{il}\right]}^{\alpha }\cdot {\left[\eta_{il}\right]}^{\beta }},\;j\in N\left(i,k\right)$$

where τ_ij_ denotes pheromone concentration, η_ij_ denotes the heuristic information provided by different methods, α and β denote the pheromone weight and heuristic weight, respectively, and N(i,k) denotes the set of candidate nodes available to the vehicle or ant in the current state. The unified framework consists of a data-generation module, a heuristic-generation module, an ACO solving module, a statistical-evaluation module, and a visualization-reporting module.

The implementation uses the complete currently feasible node set rather than a fixed-size candidate list. Pheromone is initialized to 1 on every edge. For each of 100 iterations, 30 ants sample transitions from weights proportional to $[\tau_{ij}{]}^{\alpha }\cdot [\eta_{ij}{]}^{\beta }$, with α = β = 1 in the revised main comparison. After multiplying pheromone by the retention factor 0.9, every ant deposits 1/Lk on each traversed edge; TSP deposits are symmetric, whereas CVRP deposits follow the directed route representation. No 2-opt, other local search, elitist update, or pheromone bounds are used, except for a 10^−10^ numerical floor in the CVRP pheromone matrix. TSP starts each ant at a uniformly sampled city. CVRP starts at the depot, masks visited customers and demands that exceed remaining capacity, permits depot return to reset capacity, and stops only after every customer is visited and every ant has returned to the depot. The product with the feasibility masks is subsequently floored at 10^−30^ before categorical sampling to avoid a degenerate all-zero vector.

For every method, the heuristic adapter produces an n × n raw edge-score matrix zij. Human rules and the ReEvo-offline proxy candidates return explicit positive formulas: GHPP first applies |zij| and the MLP proxy uses a Softplus output. The common evaluator then adds 10^−9^, clips to [10^−9^, 10^8^], and maps NaN, +∞, and −∞ to 10^−9^, 10^8^, and 10^−9^, respectively. GHPP and the MLP proxy additionally cap their internal outputs at 10^6^ before this common step. There is no temperature transform or rank normalization. With β = 1, multiplication of an entire positive heuristic matrix by a constant cancels during categorical normalization but additive offsets and within-row score shapes do not; this remaining adapter limitation is stated explicitly rather than assumed away.

### 3.3. Compared Methods and Parameter Settings

This paper compares four interface classes. (1) Human-designed heuristics, including inverse distance, nearest-neighbor weighting, and savings heuristics. (2) GHPP, which uses tournament selection, crossover, and mutation to generate expression rules. (3) A resource-constrained DeepACO-MLP proxy (hidden dimension 64, 20 training instances, and 50 epochs), not the GNN architecture or training scale of the original DeepACO study [10]. (4) A ReEvo-offline proxy that screens a repository-provided set of pre-specified ReEvo-style candidate rules; the LLM evolution itself is not rerun. This is therefore a controlled interface comparison, not a full-capability reproduction of every original method. Solver parameters and held-out test instances are shared, while generation and training budgets and their provenance are reported explicitly rather than claimed to be identical. The main experimental settings are summarized in Table 3.

**Table 3:** Main experimental parameter settings.

Module	Parameter	Setting
ACO solver	Number of ants, iterations, α, β, pheromone decay rate	30 ants; 100 iterations; α = 1; β = 1 for all methods; pheromone retention factor = 0.9
GHPP	Population size, evolutionary generations, maximum depth	50, 30, 6
DeepACO-MLP proxy	Hidden dimension, activation function, learning rate, training epochs	Hidden dimension 64; Softplus output; learning rate 0.001; 50 epochs; 20 training instances
ReEvo-offline proxy	Execution mode	Offline screening of pre-specified candidate rules; LLM evolution and API calls not rerun
Data replication	Number of training instances, number of test instances	20 training instances; 200 test instances for each scale

### 3.4. Statistical Testing Method

Because the same test instances would ideally support paired analysis but the instance-level objective arrays were not retained in the archived results, we use an approximate Welch test reconstructed from the reported group means, standard deviations, and n = 200 test instances. The analysis is conditional on the selected trained or generated heuristic and quantifies variation across problem instances rather than variability across independent training or evolutionary runs. For the eight prespecified comparisons, Holm’s step-down procedure controls the family-wise error rate. We report the raw *p*-values and Cohen’s d; every conclusion remains significant after Holm adjustment (pHolm < 0.001). Future work should retain per-instance paired outcomes and repeat each stochastic generation procedure across independent seeds.

(4) $$t=({\bar{x}}_{1}-{\bar{x}}_{2})/\surd (s_{1}^{2}/n_{1}+s_{2}^{2}/n_{2})$$

Significance markers are defined as follows: * indicates *p* < 0.05, ** indicates *p* < 0.01, *** indicates *p* < 0.001, and n.s. indicates no significant difference. Means and standard deviations in the main tables describe the distribution over held-out problem instances for one fixed heuristic realization; they are not multi-run training or evolution statistics.

### 3.5. Computing Environment, Reproducibility, and Timing Protocol

All archived experiments were run on one CPU-only desktop; no GPU or CUDA acceleration was used (device = ‘cpu’). The retained hardware record identifies the processor only as an Intel Core i5/i7-class or equivalent CPU and does not preserve the exact model, core/thread count, installed RAM, or operating-system build; these missing specifications are reported transparently rather than inferred. The recorded software stack is Python 3.11 (https://www.python.org/), PyTorch 2.x (https://pytorch.org/), NumPy 1.26 (https://numpy.org/), and SciPy 1.17 (https://scipy.org/) (all accessed on 17 April 2026), the archived record does not preserve the PyTorch minor/patch version. Methods, problem scales, test instances, DeepACO-MLP epochs, and training instances are processed sequentially. Within one ACO solve, the 30 ants are represented by batched PyTorch tensors, while iterations, construction steps, and pheromone updates remain sequential; no multiprocessing, instance batching, or distributed execution is used. The MLP contains 4609 TSP parameters and 4737 CVRP parameters. The principal TSP-500 ACO state tensors occupy approximately 3.18 MB, but process-level peak RSS was not instrumented and is therefore not claimed.

The experiment initializes Python, NumPy, and PyTorch with global seed 1234; GHPP search separately uses seed 42. Dataset seeds are TSP train 1234, TSP test 2234–2237 for 50/100/200/500 nodes, CVRP train 1334, and CVRP test 2334–2337 for 20/50/100/200 nodes. The ACO random stream is initialized once, not independently reseeded before every method, and deterministic-algorithm and thread controls were not enabled; reproducibility is therefore repository- and environment-level rather than a claim of cross-platform bitwise identity. Wall-clock measurements use time.time(). Generation/training time and unified-solver evaluation time are separate stages and are not silently combined when their provenance differs.

## 4. Experimental Design

### 4.1. Dataset Settings

This paper selects TSP and CVRP because they are widely used routing benchmarks, differ in feasibility structure (permutation-only versus capacity-constrained route partitioning), and can share one constructive ACO transition interface. TSP coordinates are sampled independently from the uniform distribution on [0,1]^2^. CVRP customer coordinates use the same distribution, demands are sampled from the discrete uniform distribution over the integers 1–9, the depot is fixed at (0.5, 0.5), and vehicle capacity is Q = 50. These choices define a controlled synthetic distribution for the present study; they are not asserted to reproduce every configuration in prior NCO work.

The scope is deliberately restricted to routing problems that can share an identical node-selection mechanism. Job-shop scheduling, VRPTW, and pickup-and-delivery require different state variables, action semantics, precedence or time-window masks, and repair operators; adding them without redesigning the adapter would no longer be a controlled comparison of a fixed ACO backbone. The conclusions are therefore limited to the studied nature-inspired routing setting.

Training and test sets are disjointed by seed, and no test instance is used during heuristic generation or MLP training. The protocol evaluates held-out instances and cross-scale behavior under the same uniform coordinate generator. It does not test distribution shift; accordingly, the revised manuscript uses ‘cross-scale behavior under a matched distribution’ rather than ‘out-of-distribution generalization’. Clustered and non-uniform coordinates, altered demand distributions, and TSPLIB/CVRPLIB instances are reserved for a future distribution-shift study. The TSP and CVRP dataset settings are summarized in Table 4 and Table 5, respectively.

**Table 4:** TSP experimental dataset settings.

Dataset Type	Problem Scale	Number of Instances	Purpose
Training set	50 nodes	20	Method training/evolution
Test set	50 nodes	200	Same-scale performance evaluation
Test set	100 nodes	200	Matched-distribution cross-scale evaluation
Test set	200 nodes	200	Matched-distribution larger-scale evaluation
Test set	500 nodes	200	Matched-distribution largest-scale evaluation

**Table 5:** CVRP experimental dataset settings.

Dataset Type	Problem Scale	Vehicle Capacity	Number of Instances	Purpose
Training set	50 nodes	50	20	Method training/evolution
Test set	20 nodes	50	200	Small-scale evaluation
Test set	50 nodes	50	200	Same-scale evaluation
Test set	100 nodes	50	200	Matched-distribution cross-scale evaluation
Test set	200 nodes	50	200	Matched-distribution larger-scale evaluation

### 4.2. Evaluation Metrics

The evaluation metrics include (1) solution quality, measured by average route length, where lower is better; (2) instance-level dispersion, measured by standard deviation and error bars for one fixed heuristic realization; (3) approximate comparative significance, represented by Welch *p*-values, Holm adjustment, and Cohen’s d; (4) reported one-time generation or training cost, kept separate from unified-solver evaluation time and labeled by provenance; and (5) descriptive cross-scale route-length change under the following matched generator:

(5) G(a, s_1_ → s_2_) = [M(a, s_2_) − M(a, s_1_)]/M(a, s_1_) × 100%

where M(a,s) denotes the average route length of method a at scale s. A smaller value indicates stronger performance retention as the problem scale increases.

## 5. Results

### 5.1. Performance Comparison on TSP

**Figure 1:**
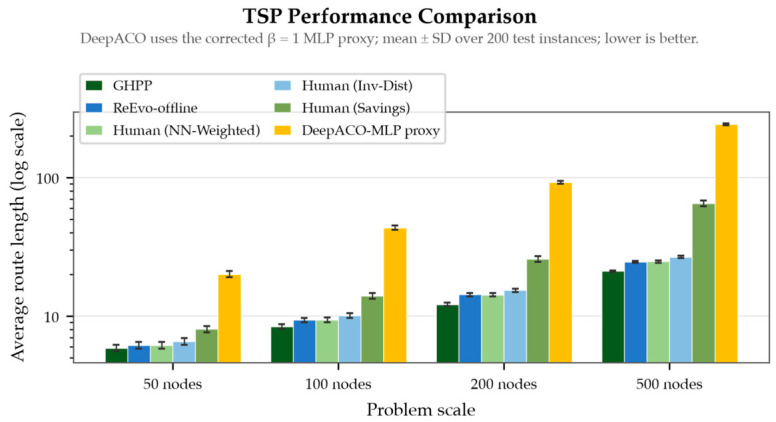
TSP route length across problem scales.

**Figure 2:**
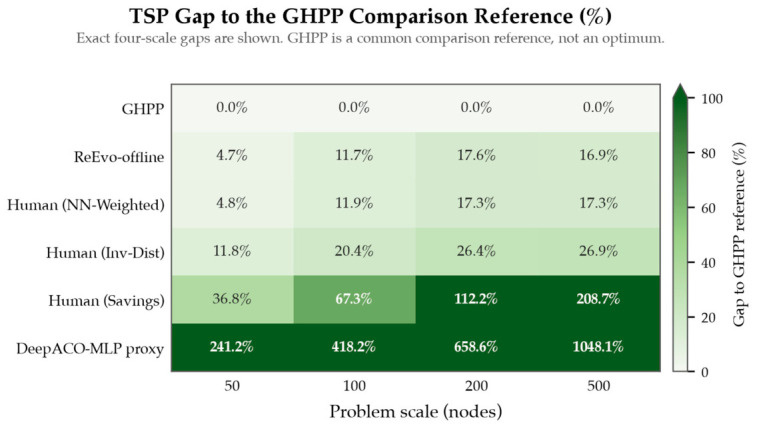
TSP relative gap versus the GHPP comparison reference.

Table 6 summarizes the TSP route-length results, while Figure 1 and Figure 2 visualize route length and relative gaps, respectively. The TSP results show that GHPP obtains the shortest routes at all four scales. ReEvo-offline and Human NNWeighted form a closely matched second tier. Their four-scale mean gaps to GHPP are 12.7% and 12.8%, respectively; the 4.7% value applies only to ReEvo-offline at TSP50 and is no longer presented as an overall average. The resource-constrained DeepACO-MLP proxy deteriorates strongly as scale increases. Against LKH near-optimal means of 5.70 and 7.78 are obtained for TSP50 and TSP100, respectively, and the gaps are GHPP +3.1%/+7.8%, ReEvo-offline +7.9%/+20.4%, Human NNWeighted +8.1%/+20.6%, and DeepACO-MLP +251.9%/+458.2%. These observations describe the evaluated proxy settings and do not rank the full original DeepACO or ReEvo systems.

**Table 6:** Performance comparison of different methods on TSP (average route length ± standard deviation). All rows use 200 held-out instances per scale; the DeepACO-MLP proxy uses the complete β = 1 record.

Method	50 Nodes	100 Nodes	200 Nodes	500 Nodes	Mean Gap vs. GHPP Over Reported Scales/Note
GHPP	5.88 ± 0.30 ***	8.38 ± 0.34 ***	12.14 ± 0.35 ***	21.07 ± 0.32 ***	0.0%; best benchmark
ReEvo-offline proxy	6.15 ± 0.34	9.36 ± 0.36	14.28 ± 0.38	24.64 ± 0.44	+12.7%; explicit-rule second tier
Human NNWeighted	6.16 ± 0.36	9.38 ± 0.38	14.24 ± 0.40	24.72 ± 0.44	+12.8%; explicit-rule second tier
Human InvDist	6.57 ± 0.38	10.09 ± 0.40	15.35 ± 0.43	26.74 ± 0.54	+21.4%; human baseline
Human Savings	8.04 ± 0.42	14.02 ± 0.65	25.77 ± 1.20	65.04 ± 3.14	+106.2%; large-scale degradation
DeepACO-MLP proxy	20.06 ± 1.02	43.43 ± 1.53	92.12 ± 2.30	241.90 ± 3.84	+591.5%; resource-constrained proxy

Note: *** indicates *p* < 0.001.

### 5.2. Performance Comparison on CVRP

**Figure 3:**
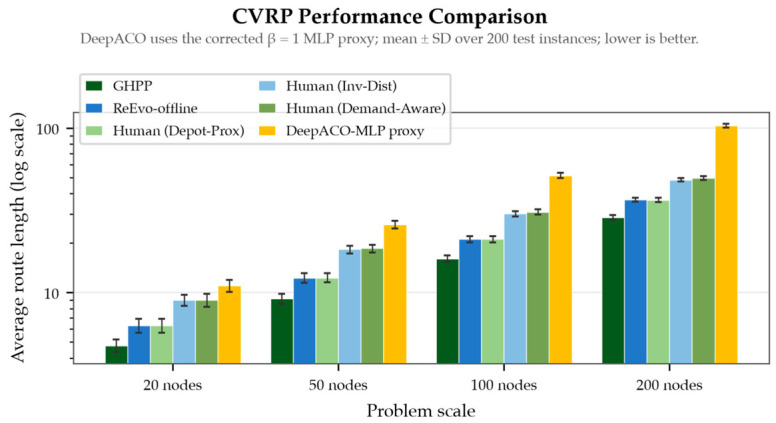
CVRP route length across problem scales.

**Figure 4:**
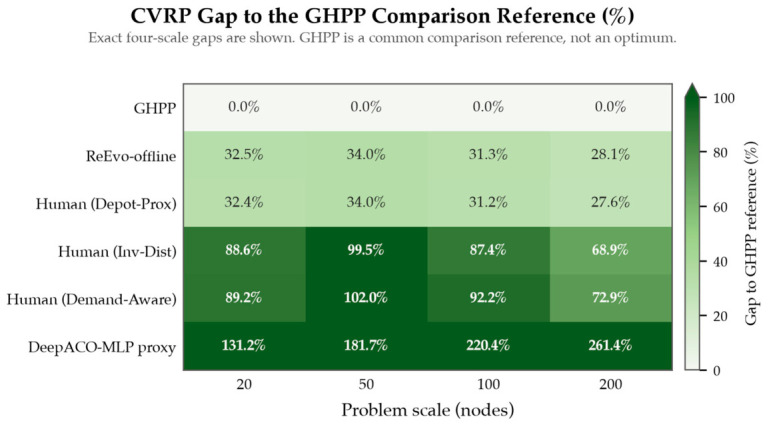
CVRP relative gap versus the GHPP comparison reference.

Table 7 summarizes the CVRP route-length results, while Figure 3 and Figure 4 provide the corresponding visual comparisons. The CVRP results show that GHPP obtains the shortest routes at all four scales. Human DepotProx and the ReEvo-offline proxy form a statistically similar second tier, with respective four-scale mean gaps of 31.3% and 31.5% to GHPP. Both outperform the resource-constrained DeepACO-MLP proxy in this implementation. Figure 3 and Figure 4 have been regenerated from the archived summary data with the revised proxy labels and corrected mean-gap interpretation.

**Table 7:** Performance comparison of different methods on CVRP (average route length ± standard deviation).

Method	20 Nodes	50 Nodes	100 Nodes	200 Nodes	Mean Gap vs. GHPP Over Reported Scales/Note
GHPP	4.76 ± 0.42 ***	9.18 ± 0.65 ***	16.08 ± 0.75 ***	28.67 ± 0.92 ***	0.0%; best benchmark
Human DepotProx	6.31 ± 0.62	12.31 ± 0.79	21.09 ± 0.90	36.57 ± 1.09	+31.3%; strong human baseline
ReEvo-offline proxy	6.31 ± 0.60	12.31 ± 0.81	21.11 ± 0.89	36.71 ± 1.05	+31.5%; explicit-rule second tier
Human InvDist	8.98 ± 0.70	18.32 ± 0.99	30.13 ± 1.06	48.42 ± 1.15	+86.1%; human baseline
Human DemandAware	9.01 ± 0.80	18.56 ± 1.02	30.91 ± 1.10	49.56 ± 1.31	+89.1%; demand-aware baseline
DeepACO-MLP proxy	11.01 ± 0.91	25.87 ± 1.38	51.50 ± 1.96	103.59 ± 2.64	+198.7%; resource-constrained proxy

Note: *** indicates *p* < 0.001.

### 5.3. Statistical Significance Testing

Table 8 shows that all eight prespecified GHPP-versus-second-tier comparisons reach raw *p* < 0.001 and remain pHolm < 0.001 after Holm’s family-wise correction. Cohen’s d values indicate large effects for every comparison. These tests use 200 held-out instances per group and are conditional on one fixed heuristic realization; they do not quantify between-run uncertainty of GHPP evolution, MLP training, or LLM generation.

**Table 8:** Welch *t*-test results for major method differences.

Task Scale	Comparison	t Value	df	*p*-Value	Cohen’s d	Significance
TSP50	GHPP vs. ReEvo-offline proxy	−8.42	391.9	7.12 × 10^−16^	−0.84	***
TSP100	GHPP vs. ReEvo-offline proxy	−27.99	396.7	6.07 × 10^−96^	−2.80	***
TSP200	GHPP vs. Human NNWeighted	−55.88	391.1	1.54 × 10^−188^	−5.59	***
TSP500	GHPP vs. ReEvo-offline proxy	−92.80	363.5	3.48 × 10^−255^	−9.28	***
CVRP20	GHPP vs. ReEvo-offline proxy	−29.93	356.3	2.92 × 10^−99^	−2.99	***
CVRP50	GHPP vs. ReEvo-offline proxy	−42.62	380.2	7.00 × 10^−147^	−4.26	***
CVRP100	GHPP vs. ReEvo-offline proxy	−61.12	386.9	7.13 × 10^−201^	−6.11	***
CVRP200	GHPP vs. ReEvo-offline proxy	−81.45	391.2	1.86 × 10^−247^	−8.14	***

Note: *** indicates raw *p* < 0.001; all eight comparisons remain significant after Holm correction (pHolm < 0.001).

### 5.4. Diagnostic Sensitivity and Provenance Checks

The archived ablation summaries are used as diagnostics of the shared ACO backbone and of subsampling within the available 20-instance training pool. They are not used to claim full method-specific hyperparameter optimization or training-data saturation. The revised interpretation separates these diagnostics from the main β = 1 comparison.

#### 5.4.1. Sensitivity of Shared ACO Backbone Parameters

**Figure 5:**
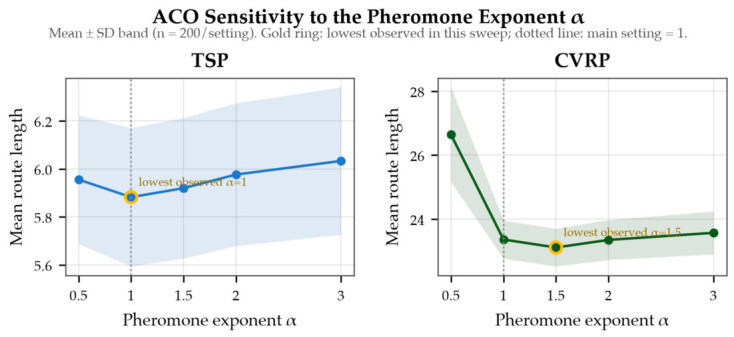
Two-panel diagnostic sensitivity of the shared ACO backbone to pheromone weight α.

**Figure 6:**
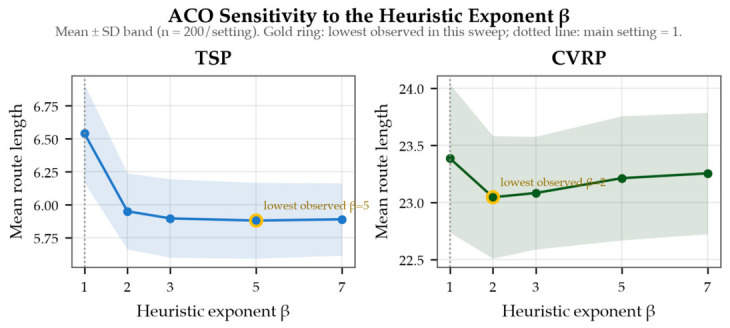
Two-panel diagnostic sensitivity of a representative explicit-heuristic ACO backbone to heuristic weight β.

**Figure 7:**
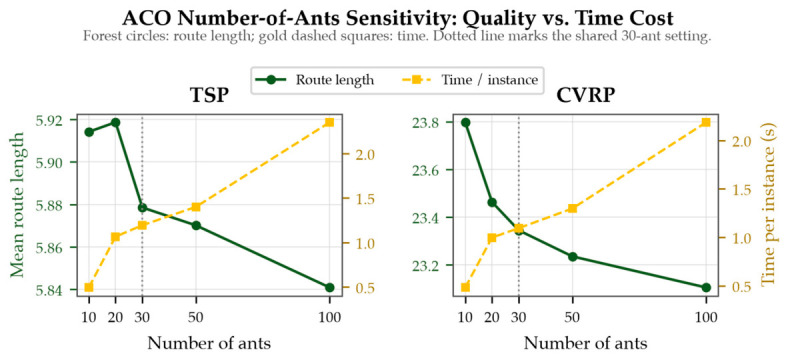
Two-panel ant-count sensitivity of the shared ACO backbone.

As the common execution backbone for different heuristic-generation methods, the ACO solver influences the final route-construction process through its parameters. To avoid systematic bias toward any method caused by a single parameter setting, this paper scans the pheromone weight α, the heuristic weight β, and the number of ants n_ants, and compares their marginal effects under fixed iteration counts and fixed test instances. The retained scan settings are summarized in Table 9; Figure 5, Figure 6 and Figure 7 visualize the corresponding sensitivity diagnostics.

Table 9 and Figure 5, Figure 6 and Figure 7 characterize sensitivity of the common ACO execution layer. The β curve is generated with a representative explicit heuristic and cannot validate a task-specific DeepACO-MLP optimum. To avoid mixing a method-specific scan with the controlled main comparison, the revised Table 3, Table 6 and Table 7 use β = 1 for all methods. The ant-count diagnostic still supports 30 ants as a quality–time compromise, while α = 1 remains within the stable region of both task summaries.

#### 5.4.2. Search-Configuration and Provenance Audit

GHPP and ReEvo-style rules have different cost provenances. The archived GHPP run locally evaluates a population of 50 for 30 generations. The present ReEvo-offline proxy does not execute LLM evolution; it only screens pre-specified candidate rules on the local training instances. Table 10 therefore reports configuration and provenance, not an unarchived budget-ablation claim.

Table 10 prevents local screening time, external LLM-generation estimates, and local GP search from being merged into one speedup claim. The revised manuscript therefore removes the inconsistent 269× value and treats the nominal 117× ratio of 585.21 min to the externally reported 5 min only as descriptive mixed-provenance context.

#### 5.4.3. Exploratory Sensitivity Within the Available Training Pool

**Figure 8:**
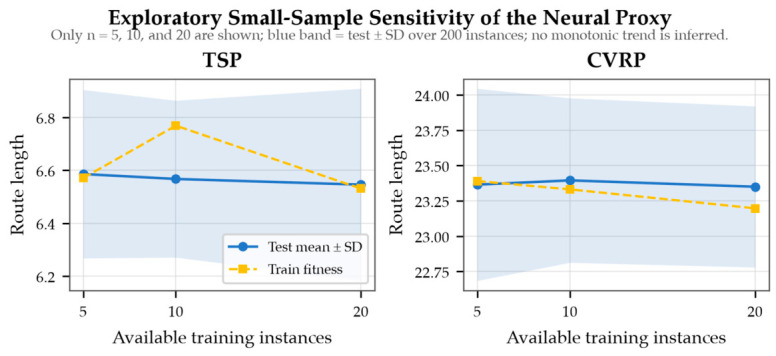
Two-panel exploratory sensitivity for 5, 10, and 20 available training instances.

The archived training pool contains 20 instances. We therefore retain only the actual 5-, 10-, and 20-instance subset conditions and treat them as an exploratory sensitivity check. The deleted 50 and 100 labels reused the same 20-instance pool and could not support a saturation claim. The retained subset analysis is summarized in Table 11 and visualized in Figure 8.

Table 11 and the regenerated Figure 8 compare only ntrain = 5, 10, and 20. Test means vary modestly within this small pool, but these data neither establish saturation at 20 nor predict behavior at 50 or 100 genuinely distinct training instances. Larger independently generated training sets and multiple training seeds are needed to separate data-volume and architecture effects.

Taken together, the archived diagnostics show that the ACO execution layer is sensitive to heuristic weighting and ant count, and that estimates within the small available training pool are reasonably stable. They do not validate a hyperparameter optimum for the original GNN-based DeepACO, a 20-instance saturation point, or the robustness of stochastic heuristic generation across seeds. The main results are therefore presented as a conditional benchmark of the archived proxy implementations.

### 5.5. Computational Cost and Interpretability Analysis

**Figure 9:**
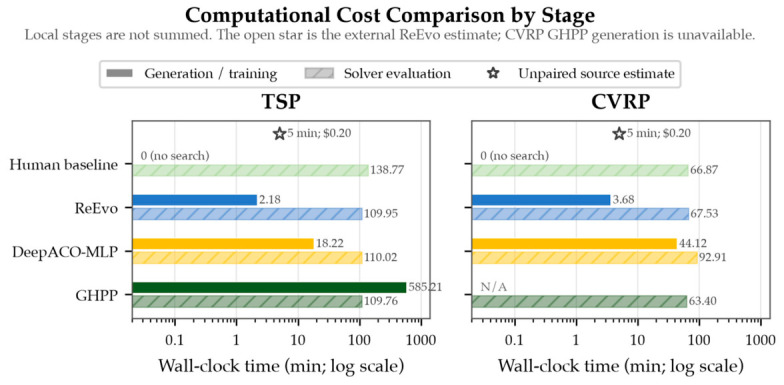
Two-panel, horizontal log-scale timing audit.

Table 12 consolidates the reported one-time and stage-separated timing evidence, and Figure 9 visualizes the stage-separated audit. Table 12 reports one-time generation or training stages, not harmonized end-to-end time. GHPP and the two DeepACO-MLP proxy training times were measured in the local CPU implementation. The 5 min and USD 0.20 values for ReEvo-offline proxy are reported in the source study [19]; the present run did not call an LLM and instead spent 2.18 min (TSP) and 3.68 min (CVRP) screening cached candidates. Consequently, 585.21/5.00 = 117.0 is only a nominal mixed-provenance ratio and is not interpreted as a controlled speedup. The former 269× value used local candidate-screening time as a different denominator and has been removed.

In terms of interpretability, the ReEvo-offline proxy candidates and human baselines expose readable Python or algebraic rules. GHPP also produces explicit expressions, although its archived high-quality result follows a long GP search. The DeepACO-MLP proxy stores heuristic information in neural-network weights and is less directly auditable. This comparison concerns representational transparency of the archived outputs, not a claim that all LLM-generated or neural heuristics have the same interpretability profile [30].

### 5.6. Cross-Scale Behavior Under a Matched Distribution

**Figure 10:**
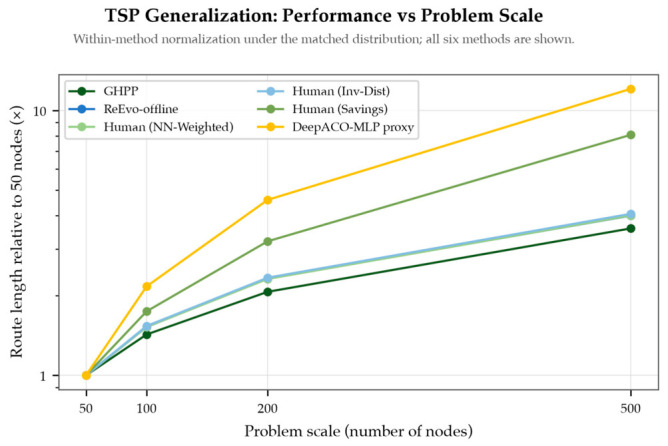
TSP cross-scale behavior under the matched synthetic distribution.

**Figure 11:**
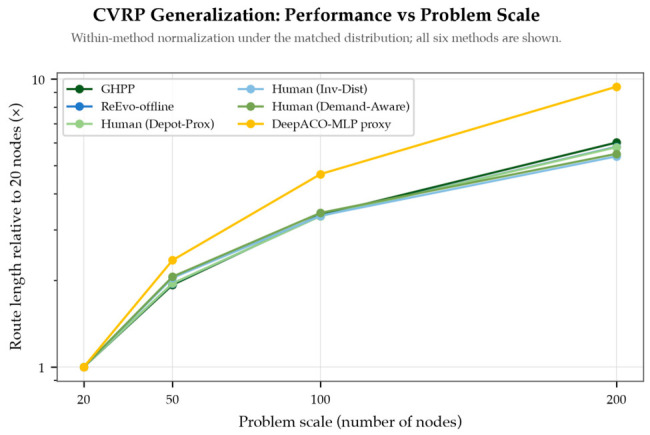
CVRP cross-scale behavior under the matched synthetic distribution.

The descriptive cross-scale changes are summarized in Table 13. Figure 10 and Figure 11 show raw route length normalized to the smallest tested scale for each method. Because route length necessarily grows with problem size, these curves are descriptive scale-response plots rather than a distribution-shift metric. The DeepACO-MLP proxy has the steepest growth in both tasks; GHPP retains the lowest absolute route length; and the explicit-rule second tier remains close within each task. All scales are sampled from the same generator.

**Table 13:** Descriptive cross-scale route-length change under the matched synthetic distribution; this is not an out-of-distribution test.

Method	TSP50 → TSP500	CVRP20 → CVRP200	Integrated Interpretation
GHPP	+258.3%	+502.3%	Best absolute quality, but highest cost; suitable for offline high-quality search.
ReEvo-offline proxy	+300.4%	+481.8%	Second-tier route quality in the archived proxy; generation-cost provenance is mixed.
Human NNWeighted/DepotProx	+301.0%	+479.6%	Human-designed rules have the lowest cost but lack automatic generation and semantic evolution capability.
Human InvDist/DemandAware	+306.8%	+450.1%	Simple baselines are interpretable, but solution quality is clearly weaker than GHPP and the ReEvo-offline proxy second tier.
DeepACO-MLP proxy	+1105.8%	+840.9%	Steepest scale-related growth in the resource-constrained proxy setting.

**Figure 12:**
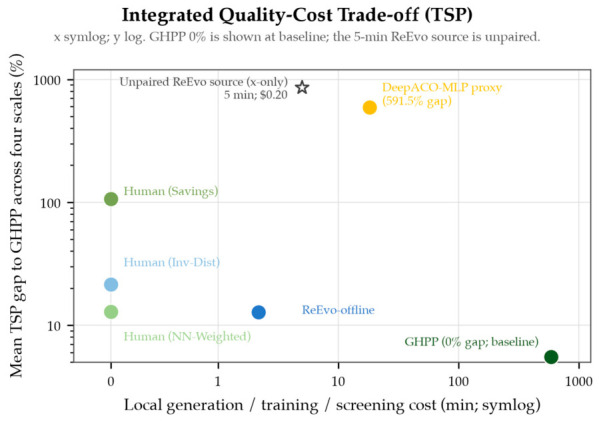
Descriptive TSP quality–cost context using corrected four-scale mean gaps.

Figure 12 uses the corrected four-scale TSP mean gaps: ReEvo-offline 12.7%, Human NNWeighted 12.8%, Human InvDist 21.4%, Human Savings 106.2%, and the DeepACO-MLP proxy 591.5%, with GHPP as 0%. The plotted ReEvo-offline x-value is the 2.18 min local cached-candidate screening stage; the externally reported 5 min generation value from [19] is shown only as an x-only, unpaired reference marker. Other non-zero x-values are local generation or training stages. The plot therefore provides descriptive context and does not establish a same-hardware Pareto frontier.

## 6. Discussion

### 6.1. Why GHPP Performs Best on TSP

The archived results show that GHPP obtains the shortest routes at every tested TSP and CVRP scale under the matched synthetic generator. The archived GHPP rule therefore remains the strongest evaluated rule across these scales, without establishing transfer to a shifted distribution. This quality comes with a 585.21 min locally measured search stage, making the setting more appropriate for offline search than for rapid, frequently repeated redesign.

### 6.2. Interpretation of the ReEvo-Offline Proxy

The evaluated ReEvo-offline proxy produces explicit, inspectable rules and places close to strong human rules, but the local experiment does not execute ReEvo’s LLM-driven evolution. Its four-scale mean gap to GHPP is 12.7% on TSP and 31.5% on CVRP. The externally reported 5 min generation value and USD 0.20 API cost provide source-study context only; they cannot be compared as a same-machine speedup against the locally measured 585.21 min GHPP search. The valid conclusion is therefore limited: cached ReEvo-style explicit rules form a competitive second tier in the shared ACO evaluator, while the generation efficiency of full ReEvo remains outside the local experiment.

### 6.3. What the Resource-Constrained DeepACO-MLP Proxy Shows—And Does Not Show

The DeepACO-MLP result must not be interpreted as an estimate of the original GNN-based DeepACO architecture [10]. It jointly reflects a reduced-capacity MLP, 20 training instances, no data augmentation, a limited training schedule, and a simplified training implementation. The experiment therefore reports the behavior of a resource-constrained neural heuristic adapter within the common interface; it neither isolates architecture from data volume nor supports a general claim that neural ACO is inferior to symbolic heuristics. A budget-parity MLP/full-GNN factorial study with genuinely larger training sets and multiple independent seeds is required to answer that causal question. This limitation is consistent with broader cautions about sample efficiency and graph-learning interfaces in combinatorial optimization [31,32,33,34].

### 6.4. Relationship with Existing Studies and Recent SOTA

Relative to prior work, the contribution here is a shared solver-interface evaluation of archived heuristic outputs rather than a new optimizer or a universal SOTA claim. Exact solvers and high-performance routing systems remain external references for absolute solution quality. Within the present experiment, human rules, GHPP expressions, a resource-constrained MLP proxy, and cached ReEvo-style rules enter the same implemented ACO route-construction and pheromone-update layer; their generation or training budgets and evidence provenance are not identical. The benchmark therefore supports a controlled comparison of route quality and representational properties within this interface, together with a stage-separated cost audit. It does not establish precise full-capability rankings across paradigms.

### 6.5. Theoretical Contributions

The contribution is a controlled interface and reporting protocol rather than a universal paradigm ranking. It places symbolic, neural-proxy, and LLM-style-proxy outputs in one ant-inspired execution layer; shows why quality, provenance-aware cost, interpretability, and matched-distribution scale response should be reported separately; and demonstrates that conclusions can change when unsupported cost denominators or full-capability labels are removed.

### 6.6. Practical Implications

For the archived settings, GHPP is the appropriate choice when route quality is prioritized and a long locally measured offline search is acceptable. Human-designed rules and the ReEvo-offline proxy are transparent second-tier baselines. No deployment recommendation is made for full ReEvo or the original GNN-based DeepACO because their original generation or training pipelines were not reproduced here. Exact OR solvers and stronger routing systems remain necessary external references when absolute solution quality is the objective.

### 6.7. Limitations and Future Work

This study has six principal limitations. (1) Instance-level objective arrays were not retained, so significance tests are approximate unpaired Welch tests reconstructed from summaries; future work should retain paired outputs and confidence intervals. (2) Stochastic GHPP search and MLP training were not repeated across independent method seeds; the reported standard deviations are across problem instances. (3) The DeepACO-MLP proxy is not the original GNN and cannot separate architecture from data volume; a multi-seed MLP/GNN × small/typical-data factorial experiment is required. (4) The local run uses cached ReEvo-offline proxy candidates and does not reproduce the LLM evolution, so external generation time and API cost remain descriptive. (5) Tests are limited to TSP/CVRP and a matched uniform generator. VRPTW and pickup-and-delivery require time-window/precedence-aware adapters, job-shop scheduling requires a different construction graph, and clustered or benchmark-library instances are needed for distribution-shift evaluation. (6) Exact CPU model, installed RAM, OS build, process-level peak RSS, and low-level thread configuration were not retained in the original run log. Future releases should archive these fields with commit hashes, dataset hashes, commands, and per-stage timing.

## 7. Conclusions

This paper presents a controlled cross-paradigm interface for evaluating heterogeneous heuristic representations within one biomimetic ACO execution layer. The revision makes the scope explicit: GHPP and human rules are locally executed baselines, the neural result is a resource-constrained DeepACO-MLP proxy, and the LLM-style result is a ReEvo-offline proxy that does not rerun LLM evolution.

Across the archived held-out instances, GHPP achieves the shortest mean routes at every tested TSP and CVRP scale. ReEvo-offline and strong human rules form a second tier, with corrected four-scale mean gaps of 12.7% (TSP) and about 31% (CVRP) relative to GHPP. All eight prespecified GHPP-versus-second-tier comparisons remain significant after Holm correction, conditional on the fixed heuristic realizations. The resource-constrained DeepACO-MLP proxy degrades strongly with scale, but this cannot be generalized to the original GNN architecture.

The defensible conclusion is not that one heuristic-generation paradigm universally dominates. Rather, a fair biomimetic benchmark must keep the ant-inspired substrate fixed, label proxy implementations precisely, separate locally measured stages from external estimates, and report uncertainty at the level actually observed. Future work will extend the feasibility adapter to richer routing constraints, test genuine distribution shift, reproduce full GNN and LLM-generation pipelines, and repeat stochastic method generation across independent seeds.

## Figures and Tables

**Table 1:** Research gaps, research questions, and the approach of this paper.

Research Gap	Existing Limitation	Research Question	Approach in This Paper
Benchmark gap	Different paradigms often use different instances, solvers, and metrics, making direct comparison difficult.	RQ1: Under the same TSP/CVRP instances and the same ACO framework, are the differences in solution quality among methods statistically significant?	Unified datasets, unified ACO parameters, and reporting of mean ± standard deviation and *p*-values.
Cost–interpretability gap	Most studies report only solution quality and rarely quantify time, API cost, and rule readability simultaneously.	RQ2: Is there a stable trade-off among computational cost, interpretability, and solution quality?	Joint evaluation of search time, API cost, rule form, and black-box degree.
Data-dependence gap	A lightweight neural proxy can be data- and architecture-limited; the original full GNN is outside the current reproduction.	RQ3: How does the archived resource-constrained MLP proxy behave within the common interface?	Report the proxy setting explicitly and avoid conclusions about the original GNN-based DeepACO.
Cross-problem/cross-scale gap	Current evidence covers only TSP/CVRP and cross-scale behavior under a matched generator.	RQ4: How stable are results across the tested routing tasks and scales, without claiming OOD transfer?	Report TSP50→500 and CVRP20→200 descriptively; reserve distribution shift and new problem classes for future work.

**Table 2:** Positioning of the methods in this paper relative to recent SOTA methods.

Method Category	Representative Methods	Main Advantage	Relationship to This Paper
Exact/traditional OR benchmarks	Concorde, HGS-CVRP	Strong solution quality; can serve as external benchmarks for TSP/CVRP.	Used to illustrate the absolute SOTA boundary; not the main heuristic generators compared in this paper.
End-to-end NCO	AM, POMO, LEHD	Fast inference after training; some models have cross-scale generalization ability.	Used as learning-based SOTA references beyond DeepACO-MLP proxy to discuss generalization and training cost.
Neural-enhanced ACO	DeepACO-MLP proxy	Retains the ACO structure while learning heuristic information.	Included in the unified experiments as the representative learning-based method.
Genetic programming hyper-heuristics	GHPP	Explicit rules, moderate cost, and good stability.	Included in the unified experiments as the representative evolutionary hyper-heuristic method.
LLM-based automated algorithm design	AEL, EoH, ReEvo-offline proxy, MEoH, FunSearch	Can generate code and rules with low manual design cost.	ReEvo-offline proxy is included in the unified experiments; the other methods are used for SOTA positioning and discussion.

**Table 9:** Ablation results for ACO parameter sensitivity.

Parameter	Scan Range	Main-Experiment Value	Sensitivity Pattern	Conclusion
α	{0.5, 1.0, 1.5, 2.0, 3.0}	1.0	For TSP, α = 1.0 achieves the lowest cost of 5.8824; for CVRP, α = 1.5 is slightly lower than α = 1.0, but the gap is small. Excessively large α strengthens pheromone positive feedback, whereas excessively small α weakens the pheromone effect.	α = 1.0 is stable across both tasks, and the main experiments retain this value.
β	{1.0, 2.0, 3.0, 5.0, 7.0}	β = 1.0 for all methods in the revised main comparison	In the representative explicit-heuristic diagnostic, the lowest TSP value occurs near β = 5 and the lowest CVRP value near β = 2; this scan is not a DeepACO-MLP architecture study.	Retain β = 1 in the main cross-method comparison; treat the scan only as a backbone-sensitivity diagnostic.
n_ants	{10, 20, 30, 50, 100}	30	Increasing the number of ants can reduce average route length, but marginal benefits diminish clearly after 30, while time cost continues to rise.	n_ants = 30 reflects a trade-off between solution quality and time cost.

**Table 10:** Search configuration and provenance of the evolutionary-style comparators.

Method	Stage	Archived Setting	Available Evidence	Interpretation
GHPP	Local GP search	Population 50 × 30; depth 6	585.21 min local	One search seed
ReEvo-offline proxy	Candidate screening	Pre-specified rule set	2.18 min TSP; 3.68 min CVRP	No local LLM evolution
ReEvo source-study context	Reported generation	max_fe = 100 context	5 min; USD 0.20 in [19]	External; not same-hardware

**Table 11:** Exploratory sensitivity within the available 20-instance training pool.

Task	n_train	Train Fitness	Test Mean ± Std	Gap/Generalization Status
TSP-50	5	6.5708	6.5858 ± 0.3184	+0.23%
TSP-50	10	6.7688	6.5672 ± 0.2965	−2.98%
TSP-50	20	6.5307	6.5457 ± 0.3632	+0.23%
CVRP-50	5	23.3899	23.3646 ± 0.6800	−0.11%
CVRP-50	10	23.3312	23.3948 ± 0.5820	+0.27%
CVRP-50	20	23.1971	23.3499 ± 0.5702	+0.66%

**Table 12:** Timing audit: (a) reported one-time heuristic generation or training cost; (b) stage-separated generation/training and unified-solver evaluation times from the archived records.

Method	Time (s)	Time (min)	Provenance	API Cost (USD)	Scope Note
Human-designed	0	0.00	No search	0.00	Solver evaluation reported separately
ReEvo-offline proxy	Not rerun	5.00 reported	Source study [19]	0.20 reported	LLM not rerun; local screening separate
DeepACO-MLP proxy (TSP)	1093.38	18.22	Local CPU training	0.00	50 epochs × 20; evaluation separate
DeepACO-MLP proxy (CVRP)	2646.97	44.12	Local CPU training	0.00	50 epochs × 20; evaluation separate
GHPP	35112.41	585.21	Local CPU search	0.00	Population 50 × 30; evaluation separate
**Method**	**TSP gen./train (min)**	**TSP eval. (min)**	**CVRP gen./train (min)**	**CVRP eval. (min)**	**Denominator/Provenance**
Human representative	0.00	138.77	0.00	66.87	NNWeighted/DepotProx; 200 × 4
ReEvo-offline proxy	5.00 reported; 2.18 screening	109.95	5.00 reported; 3.68 screening	67.53	Generation [19]; local eval.; 200 × 4
DeepACO-MLP proxy	18.22	110.02	44.12	92.91	Local; 200 × 4; TSP β = 1
GHPP	585.21	109.76	Not retained	63.40	Local; 200 × 4; CVRP search unavailable

## Data Availability

Source code, configurations, generated datasets, archived summary results, and figure assets are available at https://github.com/HaoyuanWu-A/NCO (accessed on 24 June 2026, archived revision audited at commit cc02b2d11b4dc455145f8509f8f41d8b7b2ca71f).

## Abbreviations

The following abbreviations are used in this manuscript:

Abbreviation	Definition
NCO	Neural combinatorial optimization
ACO	Ant colony optimization
TSP	Traveling salesman problem
CVRP	Capacitated vehicle routing problem
GP	Genetic programming
LLM	Large language model
GHPP	Genetic programming hyper-heuristic for path problems
